# Integrated Care Models for HIV, Diabetes and Hypertension in Sub‐Saharan Africa: A Systematic Review of Effectiveness, Implementation and Real‐World Applicability

**DOI:** 10.1002/jia2.70159

**Published:** 2026-07-05

**Authors:** Charuai Suwanbamrung, Esayas Aydiko Amele, Dereje Haile, Hien Thi Nguyen, Temesgen Anjulo Ageru

**Affiliations:** ^1^ Public Health Research Program School of Public Health Walailak University Nakhon Si Thammarat Thailand; ^2^ Excellent Center for Public Health Research (EC For PHR) Walailak University Nakhon Si Thammarat Thailand; ^3^ College of Medicine and Health Sciences Wolaita Sodo University Wolaita Sodo Ethiopia; ^4^ Public Health Faculty Can Tho University of Medicine and Pharmacy Can Tho Vietnam

**Keywords:** diabetes, health systems, HIV, hypertension, implementation science, integrated care, NCDs, sub‐Saharan Africa, systematic review

## Abstract

**Background:**

Sub‐Saharan Africa faces a syndemic of HIV and non‐communicable diseases (NCDs), particularly diabetes and hypertension. Integrated care models have been promoted to improve service efficiency and patient outcomes; however, evidence on their effectiveness, implementation and real‐world applicability remains fragmented.

**Objective:**

This systematic review aimed to assess the clinical and service delivery effectiveness of integrated HIV‐diabetes‐hypertension care models in sub‐Saharan Africa and to identify key implementation barriers and facilitators, and sustainability considerations.

**Methods:**

We conducted a systematic review of studies published between January 2016 and June 2026 following PRISMA 2020 guidelines. PubMed, Scopus and African Index Medicus were searched. Eligible studies included randomized controlled trials, observational, qualitative and mixed‐method studies evaluating integrated care models for HIV and NCDs in sub‐Saharan Africa. Data were synthesized narratively, guided by implementation science frameworks.

**Results:**

Sixteen studies from eight sub‐Saharan African countries were included. Integrated care models, such as one‐stop clinics, nurse‐led service, adherence clubs and decentralized drug distribution, were consistently associated with high retention in care for people living with HIV (generally >80%) and sustained viral suppression (> 90%). Retention for diabetes and hypertension care was lower (70%–78%), and improvements in blood pressure and glycaemic control were reported but remained inconsistent and frequently suboptimal. Integrated care models improved service delivery outcomes, including appointment adherence, continuity of care and patient acceptability. Key implementation facilitators included task‐shifting, co‐located services, leadership support and leveraging existing HIV infrastructure. Common barriers were drug stockouts for NCDs, workforce shortages, fragmented data systems, stigma and confidentiality concerns, and long waiting times. Limited economic evidence indicates that personnel costs constituted the largest share of programme expenditure. While integrated care models were feasible and acceptable in routine settings, scalability and long‐term sustainability were constrained by donor dependence, supply chain weaknesses and variable health system readiness.

**Conclusions:**

Integrated HIV‐diabetes‐hypertension care models in sub‐Saharan Africa effectively maintain HIV treatment outcomes and improve service delivery, but clinical control of NCDs remains suboptimal. Sustainable and equitable integration will require deliberate strengthening of NCD‐specific supply chains, workforce training and context‐adapted implementation strategies aligned with health system readiness. Policy and programming should support differentiated, patient‐centred integration that addresses both clinical and systemic barriers.

**Abbreviations**
CHWs: community health workers
DM: diabetes mellitus
HIV: human immune deficiency virus
HTN: hypertension
JBI: Joanna Briggs Institute
LMICs: low‐ and middle‐income countries
PICOS: Populations, Intervention/Exposure, Comparator, outcomes, Study
PRISMA: Preferred Reporting Items for Systematic Reviews and Meta‐Analysis
SSA: sub‐Saharan Africa

## 1 Introduction

Sub‐Saharan Africa (SSA) faces a complex and growing public health challenge characterized by the enduring burden of HIV alongside a rapid rise in non‐communicable diseases (NCDs), particularly diabetes and hypertension [1]. This convergence has created a syndemic in which these conditions coexist, interact and exacerbate health outcomes, straining already fragile health systems designed primarily for acute and infectious disease care [2]. In response, integrated care models, which combine the prevention, diagnosis, treatment, and management of HIV, diabetes and hypertension within a single service delivery framework, have been advocated as a pragmatic strategy to improve efficiency, accessibility and patient outcomes [3].

Previous studies and pilot programmes suggest that integration can reduce duplication, lower patient costs, decrease stigma and improve retention in care [4, 5]. Several models have emerged across SSA, including one‐stop clinics, decentralized drug distribution clubs, task‐shifting nurse‐led and integrated chronic disease clinics embedded within existing HIV platforms [6, 7]. However, the implementation of these models is highly variable and influenced by contextual factors such as workforce capacity, supply chain stability, financing and health policy environments [8, 9].

Despite increasing intervention studies and qualitative evaluations, the evidence remains fragmented. Prior syntheses have largely treated clinical effectiveness and implementation processes as separate domains, lacking an integrated analysis that explains how the implementation contexts influence effectiveness [10]. Moreover, prior syntheses have frequently omitted the perspectives of patients, providers and policymakers regarding the acceptability and feasibility of integration, a critical omission for translating evidence into sustainable practice [11]. There is also a lack of systematic insight into real‐world applicability of integrated care models, that is their scalability across diverse SSA settings, and alignment with national health priorities.

Thus, a significant gap exists: a comprehensive, mixed‐evidence synthesis that concurrently examines the effectiveness of integrated HIV/diabetes/hypertension care on clinical and service outcomes, the implementation strategies and contextual barriers or facilitators influencing delivery, and the real‐world applicability of the models across heterogeneous SSA contexts. This review aims to fill that gap by systematically integrating quantitative, qualitative and mixed‐methods evidence to inform policy, practice and future research in sustainable chronic disease management in SSA. The specific objectives of the review: (1) to evaluate the clinical and service delivery effectiveness of integrated care models for HIV, diabetes and hypertension in SSA, by synthesizing outcomes; (2) to identify and describe the core components of implemented integrated care models; (3) to analyse barriers and facilitators influencing the implementation of integrated care; (4) to assess the real‐world applicability and sustainability of integrated care models.

## 2 Methods

### 2.1 Study Design and Protocol Registration

This systematic review was conducted in accordance with the Preferred Reporting Items for Systematic Review and Meta‐Analyses (PRISMA) 2020 guidelines. The review protocol was registered prospectively in PROSPERO (CRD420251271219). The review aimed to synthesize evidence on the effectiveness, implementation characteristics and real‐world applicability of integrated care models for HIV, diabetes and hypertension in SSA.

### 2.2 Eligibility Criteria

Eligibility criteria were defined using the Population‐Intervention‐Comparator‐Outcomes‐Study design (PICOS) framework.

- **Population**: Adults and adolescents in SSA living with HIV, diabetes and or hypertension, or multimorbidity involving these conditions.
- **Intervention**: Integrated care models delivering HIV and NCD services within a coordinated framework, including but not limited to one‐stop clinics, primary healthcare (PHC)‐based, decentralized drug distribution, adherence clubs, task‐shifted care or nurse‐led care.
- **Comparator**: Standard or non‐integrated care, vertical disease‐specific services or pre‐post intervention comparison where no control group is available.
- **Outcomes**: Primary: retention in care, HIV viral suppression, blood pressure control, glycaemic control (HbA1c) and medication adherence. Secondary outcomes included service delivery outcomes (e.g. appointment adherence, continuity care), patient and provider acceptability, stigma reduction, costs and sustainability indicators were reported.
- **Study design**: Randomized controlled trials, cohort studies, cross‐sectional studies, qualitative studies and mixed‐methods evaluations were eligible for inclusion.

### 2.3 Exclusion Criteria

Studies were excluded if they:

- Were conducted outside of SSA
- Those evaluating single diseases or fully vertical programmes without integration
- Did not report relevant clinical delivery, or implementation outcomes
- Were review articles, editorials, commentaries, protocols and conference proceedings.

### 2.4 Information Sources and Search Strategy

We systematically searched PubMed, Scopus and African Index Medicus for studies published between January 2016 and June 2026. The strategy combined controlled vocabulary terms (MeSH terms) and free‐text keywords related to HIV, diabetes, hypertension, integrated care, PHC and SSA. Reference lists and relevant studies were hand‐searched in Google Scholar to identify additional eligible studies. The full strategies for all databases are provided in Supplementary Material 1. The search was updated to June 2026.

### 2.5 Study Selection

All identified records were imported into EndNote 20 software, and duplicates were removed. Two reviewers independently screened titles and abstracts against the eligibility criteria. Full texts of potentially relevant studies were retrieved and assessed independently by the same reviewers. Any disagreement was resolved through discussion or consultation with a third reviewer. The study selection process was documented using a PRISMA flow diagram.

### 2.6 Data Extraction

Data were extracted independently by two reviewers using a pre‐piloted standard data extraction tool developed in Microsoft Excel. The primary and secondary outcomes for which data were sought are defined in the eligibility criteria (Section 2.2). Discrepancies in extracted data were resolved through discussion with a third reviewer.

### 2.7 Risk of Bias and Methodological Quality Assessment

The methodological quality and risk of bias of included studies were assessed independently by two reviewers using standardized tools for each study design. Disagreements were resolved through discussion with a third reviewer. The following tools were applied

- **Cross‐sectional designs**: The Joanna Briggs Institute (JBI) Critical Appraisal Checklist for Analytical Cross‐Sectional Studies.
- **Cohort studies**: The Joanna Briggs Institute (JBI) Critical Appraisal Checklist for Cohort Studies.
- **Qualitative studies**: The Critical Appraisal Skills Programme (CASP) Qualitative Checklist.
- **Mixed‐methods studies**: The Mixed‐Methods Appraisal Tool (MMAT).
- **Randomized controlled trials (RCTs)**: The Cochrane Risk of Bias 2 (RoB 2) tool of randomized controlled trials.

Each study was assigned an overall rating of low, moderate or high risks of bias based on the consensus across appraisal domains.

### 2.8 Data Synthesis

Due to substantial heterogeneity in study designs, integrated care models and outcome measures, a meta‐analysis was not conducted. Instead, findings were synthesized narratively. Studies were grouped by type of integrated care model and level of health system delivery (community, primary or facility‐based). Clinical effectiveness, service delivery outcomes, implementation barriers and facilitators, and sustainability considerations were summarized descriptively. Interpretation of implementation findings was informed by the Consolidated Frameworks for Implementation Research (CFIR).

### 2.9 Reporting and Ethics

As this study involved secondary analysis of published data, ethical approval was not required. Findings are reported in accordance with the PRISMA 2020 guidelines.

## 3 Results

### 3.1 Study Selection

The database search identified 1516 records. After removal of 730 duplicates, 786 unique records were screened by title and abstract. Of these, 54 full‐text articles were assessed for eligibility. Thirty‐eight studies were excluded due to irrelevant outcomes (*n* = 10), inappropriate study design (*n* = 24) or other reasons (*n* = 4). An updated search conducted in June 2026 identified no additional eligible studies.

**FIGURE 1:**
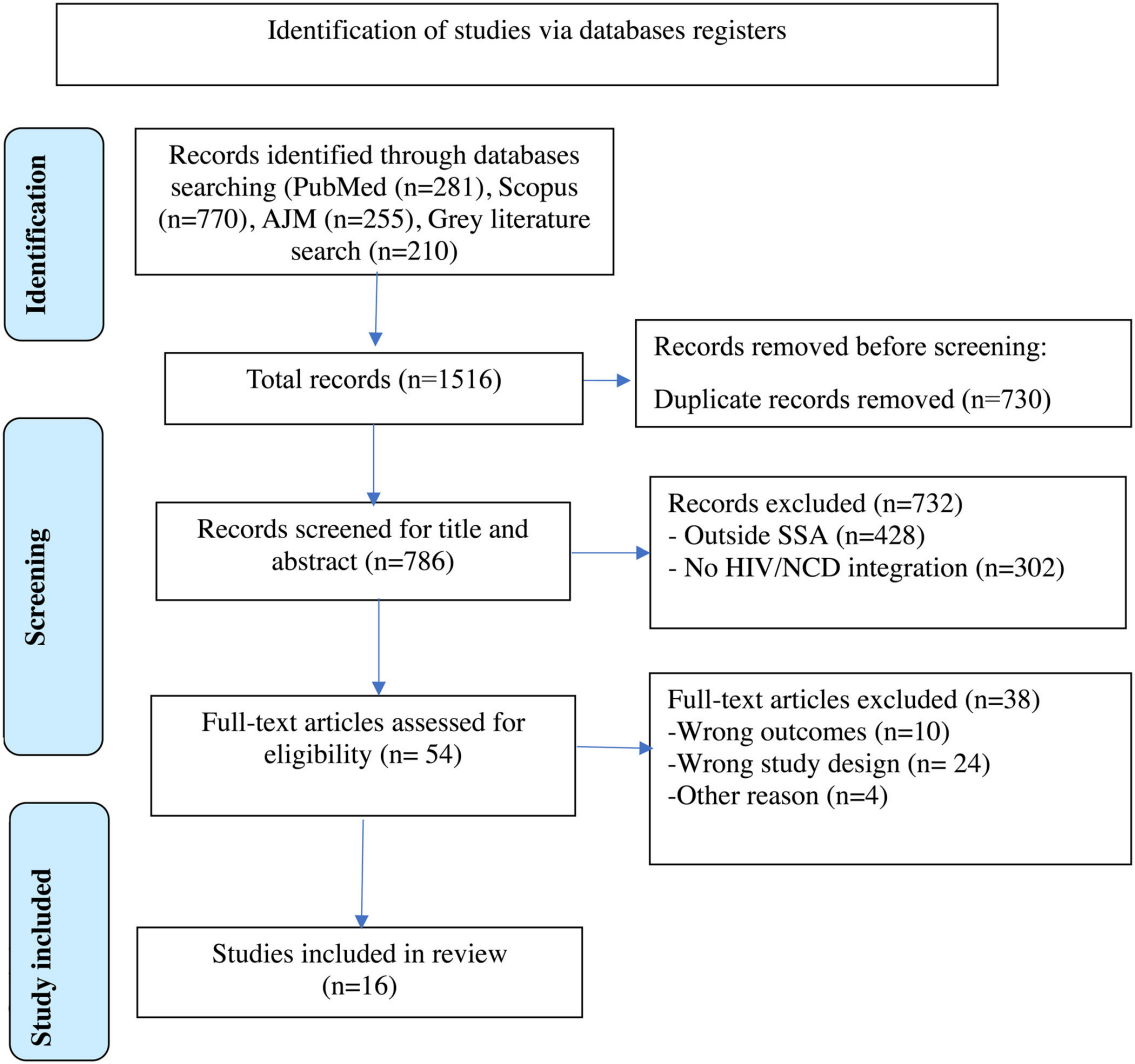
PRISMA 2020 flow chart displays the article identification, selection, screening and synthesis of included studies. *Note*: An initial search was conducted in May 2025. An updated search was performed in June 2026 to identify any newly published studies. No new eligible studies were identified beyond the 16 included in this review.

A total of 16 studies met the inclusion criteria and were included in the final synthesis. The study selection process is illustrated in the PRISMA 2020 flow diagram (Figure 1).

### 3.2 Characteristics of Included Studies

The 16 included studies were published between 2016 and 2026 and conducted across eight sub‐Saharan African countries: South Africa (*n* = 6), Tanzania (*n* = 4), Zimbabwe (*n* = 2), Ethiopia (*n* = 1), Kenya (*n* = 1), Malawi (*n* = 1) and Eswatini (*n* = 1). Three studies were multi‐country evaluations: one conducted in Tanzania and Uganda, another in Eswatini and South Africa.

The study design was diverse: randomized controlled trial (*n* = 1), cohort studies (*n* = 5), mixed methods (*n* = 3), cross‐sectional studies (*n* = 4), qualitative studies (*n* = 2) and process evaluation (*n* = 1). Sample sizes ranged from 35 to 111,163 participants, with a median sample size of approximately 415.

Most studies focused on the adult population. One study included both adults and children, with approximately 10% of participants aged under 15 years. Participants included people living with HIV only, diabetes only, hypertension only or combinations thereof (HIV + diabetes, HIV + hypertension, HIV + diabetes + hypertension). Most studies focused on adults with multimorbidity involving HIV and at least one NCD.

Eight studies described full integration, characterized by co‐located services, unified staffing and shared patient tracking. Five studies reported partial integration, typically involving coordinated referrals or added NCD services within HIV platforms. Follow‐up duration ranged from 6 to 36 months in intervention studies, while cross‐sectional studies did not report follow‐up periods (Table 1).

**TABLE 1:** Characteristics of included studies on integrated HIV, diabetes and hypertension in sub‐Saharan Africa.

Study	Country	Study design	Sample size	Population description	Condition integrated	Duration (months)
[4]	Tanzania, Uganda	RCT	7024	Adults with HIV, diabetes and hypertension (all three conditions)	HIV+ DM+ HTN	12
[12]	Zimbabwe	Mixed‐methods	305	Adults living with HIV and diabetes (without hypertension)	HIV+ DM	6
[13]	South Africa	Comparative case/cost analysis	5000	Adults with HIV and at least one NCD (diabetes and or hypertension)	HIV+ DM and or HTN (mixed)	NR
[14]	Tanzania, Uganda	Cohort study	2322	Adults with HIV and at least one NCD (diabetes and or hypertension)	HIV+ DM and or HTN (mixed)	8
[15]	Tanzania	Mixed‐methods	64	Patients and healthcare workers in integrated clinics (HIV+DM+HTN)	HIV+DM+HTN	10
[16]	Uganda	Process evaluation	63	Patients, providers, policymakers, community leaders (HIV+DM+HTN)	HIV+DM+HTN	12
[7]	Eswatini, South Africa	Cohort	111,163	Patients with HIV and or diabetes and or hypertension (any combination)	HIV and/or DM and/or HTN	24
[8]	Tanzania	Qualitative	35	Healthcare providers and managers (HIV+DM+HTN)	HIV+DM+HTN	NR
[17]	South Africa	Cohort	247	Adults with HIV and comorbid hypertension/diabetes (either condition)	HIV+HTN or HIV + DM	12
[6]	South Africa	Mixed‐methods	415	Adults with HIV and/or hypertension	HIV+HTN only	30
[18]	Ethiopia	Cross‐sectional	40	Adults living with HIV and diabetes	HIV+DM only	NR
[19]	South Africa	Cohort	4358	Adults aged ≥40 years with HIV and/or diabetes	HIV+ DM only	NR
[20]	South Africa	Cross‐sectional	301	Adults living with HIV and diabetes and/or hypertension	HIV+ DM and/or HTN (mixed)	NR
[21]	Zimbabwe	Cross‐sectional	3094	Patients with DM and/or HTN in high HIV prevalence setting	HIV+DM and/or HTN (mixed)	36
[22]	Kenya	Qualitative	106	Adults living with HIV/NCDs (diabetes and/or hypertension) and healthcare workers	HIV+ DM and/or HTN (mixed)	NR
[23]	Malawi	Cohort	6233	Adults living with HIV and diabetes (multimorbidity)	HIV+DM only	12

Abbreviations: DM, diabetes mellitus; HTN, hypertension; NCDs, non‐communicable diseases; NR, note reported.

#### 3.2.1 Definitions of Integration

**Full integration**: This term was defined as a model in which HIV, diabetes and hypertension services are provided to both HIV‐positive and HIV‐negative clients within the same chronic care clinic, using unified staffing, shared patient records, integrated medication dispensing and multidisciplinary teams.

**Partial integration**: This integration is defined as NCD services added onto existing HIV vertical clinics, where the HIV platform remains the dominant framework, and HIV‐negative clients with NCDs are generally not included.

### 3.3 Risk of Bias Assessment

Of the 16 included studies, 10 were rated as having low risk of bias and six as moderate risk. No studies were excluded due to high risk of bias. Detailed assessments for each study, including domain‐specific ratings, are provided in File S2.

### 3.4 Clinical and Service Delivery of Integrated Care Models

Overall, integrated care models for HIV, diabetes and hypertension were associated with positive service delivery outcomes, although clinical outcomes for NCDs were less consistently reported and more variable (Table 2).

**TABLE 2:** Clinical and service delivery effectiveness of integrated care models of HIV, diabetes and hypertension in sub‐Saharan Africa.

Study	Integrated model type	HIV outcomes	Diabetes outcomes	Hypertension outcomes	Service delivery outcomes	Key findings
[4]	Integrated PHC model (HIV‐DM‐HTN)	Viral suppression: 90%, Retention in care: 85%	HbA1c <7% in 75% of patients	BP control: 80%	High retention in care and continuity of care	Integrated care achieved high retention and improved outcomes across conditions
[14]	One‐stop integrated clinic	Retention: 82.5%	Retention: 85%; HbA1c <7% in 70%	Retention: 84.6%; BP control: 78%	Improved appointment adherence	High retention in care across HIV, diabetes and hypertension
[15]	Integrated HIV‐NCD clinic	Not reported	Not reported	Not reported	High acceptability among patients	Integrated services were acceptable and patient‐centred
[13]	Integrated chronic care model	Retention: 88%	Not reported	Not reported	Reduced duplication of visits	Integrated care was cost‐effective and feasible
[17]	Integrated chronic care clinic	Adherence: 95%	Not reported	BP control: 85%	Retention: 93.2%	Integrated care improved adherence and blood pressure outcomes
[6]	Integrated NCD‐HIV care	Not reported	Not reported	BP reduction: 10 mmHg	Improved follow‐up	Integration led to BP improvement
[21]	Nurse‐led integrated care	Viral suppression: 93%	Not reported	Not reported	Task‐shifting effective	Nurses successfully delivered integrated care
[16]	Integrated HIV‐NCD care	Viral suppression: 92%	Not reported	Not reported	Improved service uptake	Integration enhanced early detection and care engagement
[7]	Decentralized integrated model	Viral suppression: 89%	Not reported	BP monitoring: 82%	Reduced missed appointments	Decentralization improved continuity of care
[22]	Integrated medication adherence clubs	Viral suppression: 95%	Not reported	Not reported	High retention in care and acceptability	Integrated clubs improved continuity and satisfaction
[20]	Integrated clinic‐based care	Retention: 90%	Not reported	Not reported	Improved care coordination	Integration feasible within routine services
[19]	Integrated care model	Viral suppression: 85%	Not reported	Not reported	Comparable retention in care	Comorbidity associated with poorer HIV outcomes

#### 3.4.1 HIV Outcomes

Across models, HIV treatment outcomes were largely maintained or improved following integration. Retention in HIV care was generally high, exceeding 80% in most studies, and viral suppression rates consistently exceeded 90% in settings where these outcomes were reported. These findings were observed across diverse models including one‐stop clinics, nurse‐led services, adherence clubs and decentralized drug distribution systems. Notably, studies embedding NCD services into vertical HIV platforms (partial integration) consistently reported stronger HIV outcomes (retention >85%, viral suppression >90%) but more variable NCD control (retention: 70%–78%, control rates of <60%). In contrast, studies evaluating true chronic care clinics serving both HIV‐positive and HIV‐negative clients (full integration) reported more balanced outcomes across conditions, though fewer such studies were available and sample sizes were smaller.

#### 3.4.2 Diabetes and Hypertension Outcomes

Evidence for diabetes and hypertension outcomes was more limited. Where reported, retention in NCD care ranged from approximately 70% to 78%. Improvements in glycaemic and blood pressure control were observed in some studies; however, control rates were frequently maintained below recommended targets, and reporting of NCD‐specific clinical indicators was inconsistent across studies.

#### 3.4.3 Service Delivery Outcomes

Integrated care models were consistently associated with improvements in service delivery, including reduced duplication of visits, improved appointment adherence and high levels of patient acceptability. Several studies also reported improved continuity of care and reduced missed appointments following integration (Table 2).

### 3.5 Core Components of Integrated Care Models

Integrated HIV‐diabetes‐hypertension care models shared several core components, with variation in the degree of integration (Table 3). Fully integrated care models, such as one‐stop clinics and national integration programmes, typically featured co‐located services, unified patient records, integrated medication dispensing and multidisciplinary teams.

**TABLE 3:** Core components of integrated HIV, diabetes and hypertension care models in sub‐Saharan Africa.

Study	Integrated model type	Level of service integration	DSD: WHEN (visit/refill)	DSD: WHERE (service location)	DSD: WHO (provider type)	Medication and supply	Health information system	Community/patient support
[4, 14]	One‐stop integrated clinic	Full (serves HIV+ and HIV− clients with NCDs)	Monthly to quarter visits; 3‐month refills	Dedicated chronic care clinic at PHC/district	Multidisciplinary teams (clinicians, nurses, pharmacists)	Unified drug dispensing; integrated supply chain	Single shared patient record (electronic)	Adherence counselling ; appointment reminders
[13, 20]	Integrated HIV‐NCD clinics within existing services	Partial (HIV platform with NCD add‐on)	HIV visits per national guidelines; NCD clinical monitoring (e.g. BP, HbA1c) was less frequent; Visit frequency for refills was similar	HIV clinic within PHC facility (general OPD queue for NCDs)	HIV clinic staff with added NCD roles	Shared HIV pharmacy; limited NCD stock; separate NCD supply chain	HIV‐centred records; NCD data often missing	Minimal structured support
[17, 21]	Nurse‐led/task‐shift integrated care model	Partial (task‐shifted NCD care within HIV platform)	Aligned HIV and NCD visits (monthly to quarterly)	PHC facility (dedicate chronic care room)	Nurse‐led with physician supervision; CHWs	Integrated medication refills (same visits)	Routine clinic records (paper‐based often)	Counselling and follow‐up by nurses
[7, 22]	Decentralized community‐linked integration model	Partial (community‐based pickup)	Quarterly to 6‐month refills; clinical visits every 6 months	Community pick‐up points; adherence clubs; home visits	Nurse, CHW, lay workers, peer supporters	Community‐based medication pick‐up; centralized distribution hub	Clubs/community registers; separate from clinic records	Peer support groups; reduced travel burden
[6, 12, 18]	PHC‐based integration model	Hybrid (mixed integration within general PHC)	Variable; often NCD visits less frequent than HIV	General PHC clinic	PHC nurses and clinicians (rotating staff)	Partial or inconsistent supply chains; frequent NCD stocks	Fragmented data systems; no unified record	Health education only; no structured support
[8]	National or policy‐driven integration programme	Full (system‐wide, serves all clients)	Standardized aligned visits	PHC, district and tertiary levels	National and facility staff	Centralized procurement; integrated supply chains for all commodities	National HIS with HIV and NCD modules	Community sensitization; national campaigns

Abbreviations: DSD, differentiated service delivery; HIV, human immunodeficiency virus; NCD, non‐communicable disease; OPD, outpatient department; PHC, primary healthcare.

Partial integrated care models, often embedded within existing HIV or PHC platforms, relied on HIV‐focused workflows with added NCD service. Task‐shifting and nurse‐led care were common across models, supported by simplified clinical algorithms. Fragmentation in health information systems and incomplete integration of NCD medication supply chains were frequently reported, particularly in PHC‐based and HIV platform models (Table 3).

In partial integration models [13, 20], while medication refill visit frequency was often aligned between HIV and NCDs, the intensity of clinical monitoring (e.g. blood pressure measurement, HbA1c testing) was frequently lower for NCDs due to limited equipment, testing availability and HIV‐dominant clinical workflows.

### 3.6 Barriers and Facilitators to Implementation

Implementation of integrated care models was influenced by barriers and facilitators operating at multiple levels (Table 4). Facilitators included co‐located services, task‐shifting to nurse and community health workers, leadership support, stakeholder engagement and the use of existing HIV programme infrastructure.

**TABLE 4:** Barriers and facilitators to implementation of integrated HIV, diabetes and hypertension in sub‐Saharan Africa.

Study	Themes	Facilitators	Barriers	Level of barrier/facilitator	Implementation implications
[4, 14]	One‐stop integrated clinic	Co‐located services; reduced patient visits; strong facility leadership	High patient volume; increased staff workload; limited NCD training	Clinic/health system	Effective but requires workforce strengthening
[13, 20]	Integrated HIV‐NCD clinics within existing services	Use of established HIV infrastructure; patient familiarity	Competing clinical priorities; HIV dominant workflow	Clinic/health system	Feasible but disadvantageous to NCD care without deliberate balancing
[17, 21]	Nurse‐led/task‐shift integrated care model	Task‐shifting; nurse autonomous; simplified protocols	Limited training opportunities; supervision gaps	Individual/clinic	Highly suitable for resource‐limited settings
[7, 22]	Decentralized community‐linked integration model	Community proximity; peer support; reduced travel burden	Supply chain interruptions; CHW workload	Community/health system	Strong potential for retention in care and continuity
[6, 12, 18]	PHC‐based integration model	PHC platform availability; patient acceptability	Resource constraints; weak governance; fragmented data system	Clinic/health system/policy	Implementation success depends on the system strengthening
[8]	National or policy‐driven integration programme	National policy endorsement; system‐wide guidance	Donor dependence; coordination challenges	Policy/health system	Scale‐up feasible with sustainable financing

Abbreviations: CHW, community health worker; PHC, primary healthcare.

Commonly reported barriers included NCD medication stockouts, increased staff workload, limited NCD‐specific training, fragmented data systems, stigma and confidentiality concerns, and long waiting times. Partial integrated care models were particularly affected by competing clinical priorities and HIV‐dominant workflows, which limited the consistency of NCD service delivery.

### 3.7 Real‐World Applicability, Cost and Sustainability

Evidence on real‐world applicability and sustainability indicated that integrated care models embedded within existing health system platforms, such as HIV clinics, PHC facilities and community‐based services, were more feasible to implement in routine settings (Table 5).

**TABLE 5:** Real‐world applicability and sustainability of integrated HIV, diabetes and hypertension in sub‐Saharan Africa.

Study	Integrated care models	Evidence of real‐world applicability	Cost/resources consideration	Health system and policy fit	Sustainability assessment
[13, 20]	Integrated HIV‐NCD clinics within existing services	Feasibility using existing HIV clinic infrastructure	Cost‐efficient due to shared service	Strong fit with national HIV programmes	Moderate sustainability; reliant on HIV funding streams
[7, 22]	Decentralized community‐linked integration model	Improved access and continuity through community delivery	Reduced patients transport costs; fewer facility visits; resilience on CHWs and lay workers	Supports decentralization and differentiated service delivery	High sustainability where community systems are supported
[17, 21]	Nurse‐led/task‐shift integrated care model	Effective in facilities with limited physician availability	Lower personnel costs due to task‐shifting; reduced need for specialist staff, training required	Consistent with HRH and task‐shifting policies	High sustainability with ongoing training and supervision
[12, 18]	PHC‐based integration model	Applicable across PHC settings but variable uptake	Resource constraints reported (staffing, diagnostics); no dedicated funding	Aligned with PHC strategies	Limited sustainability without financing and government support
[8]	National or policy‐driven integration programme	Demonstrated system readiness for larger‐scale implementation	Centralized procurement and financing; donor and government funding required	Strong alignment with national policies	Sustainable contingent on long‐term financing
[4, 14]	One‐stop integrated clinic	Implemented within routine public facilities with high retention in care	Reduced patient visits frequency: shared staffing and infrastructure	Aligned with PHC and chronic care frameworks	High short‐term sustainability: staffing workload noted

Nurse‐led and decentralized models demonstrated potential advantages in resource‐limited settings by reducing personnel and patient‐related costs. However, economic evidence was limited, with few studies conducting formal cost analyses. Where reported, personnel cost constituted the largest share of programme expenditure [13]. From the client perspective, integrated models reduced transport costs and time off work, with Venables et al. [22] reporting average savings of 5–10 USD per visit and Goldstein et al. [7] noting reduced visit frequency from monthly to quarterly in decentralized models. No studies conducted full cost‐effectiveness analyses including both health system and societal costs.

Long‐term sustainability was frequently constrained by dependence on donor funding, limited financing for NCD services, and weaknesses in supply chain and health system capacity. National or policy‐driven integration programmes showed potential for scale‐up but remained contingent on sustained political commitment and financing.

Among the 38 excluded studies, the most common reasons for exclusion were: no integration (*n* = 12), no relevant outcomes for both HIV and NCDs (*n* = 10), and inappropriate study design (*n* = 24). Notably, several excluded studies measured “process outcomes” (e.g. whether blood pressure was measured at all, whether HbA1c was ordered) rather than clinical control. This highlights a gap: many routine settings do not consistently perform even basic NCD evaluations, suggesting that future integration research should first focus on adherence to essential monitoring before expecting optimal control rates.

## 4 Discussion

This systematic review synthesized evidence from 16 studies across SSA to evaluate the effectiveness, implementation and real‐world applicability of integrated care models for HIV, diabetes and hypertension. Overall, the findings demonstrate that integration can successfully maintain HIV treatment outcomes and improve service delivery; however, clinical control of NCDs remain inconsistent, and sustainability challenges persist.

### 4.1 Effectiveness of Integrated Care Models

Across diverse integrated care models, high retention in HIV care and sustained viral suppression were consistently observed, with most studies reporting retention in care rates above 80% and viral suppression exceeding 90% [4, 14, 23]. These findings reinforce evidence that leveraging established HIV platforms and adopting patient‐centred service delivery approaches can maintain core HIV outcomes while expanding access to chronic care.

In contrast, outcomes of diabetes and hypertension were more variable. Integrated care approaches were associated with improved NCD retention in care (70%–78%), but glycaemic and blood pressure control rates were substantially lower, often remaining below 50%–60% of target thresholds [14, 17]. This suggests that integration improves engagement and continuity but does not automatically ensure optimal clinical control, which requires additional elements such as medication titration protocols and access to laboratory monitoring.

Paradoxically, in some integrated models, NCD monitoring remained less frequent than HIV monitoring due to: (1) HIV‐dominant clinical workflows where viral load monitoring was prioritized; (2) limited availability of blood pressure equipment and HbA1c testing; and (3) provider uncertainty about NCD treatment algorithms. Thus, physical integration did not automatically equalize monitoring intensity.

Access to HbA1c testing was limited in most routine settings, constraining optimal diabetes monitoring. Regarding hypertension, few studies explicitly referenced the 2021 WHO guideline recommending initial dual combination therapy (preferably single‐pill combination) with monthly titration until control [24].

### 4.2 The “HIV‐First” Integration Pathway and Equity Concerns

A key finding emerging from the review is the persistence of an “HIV‐first” integration pathway. Despite structural integration, HIV services consistently outperformed NCD services in terms of retention in care, monitoring and continuity [7, 8]. Reports of NCD medication stockouts and reliance on HIV‐dominant workflows indicate that integration has often occurred by extending HIV platforms without equivalent investment in NCD‐specific resources [8, 18].

While pragmatic in resource‐constrained settings, this approach risks perpetuating inequities within integrated care models, effectively creating a two‐tier system where NCD management remains secondary. Future integration efforts must, therefore, move beyond leveraging HIV infrastructure alone and explicitly prioritize equitable resourcing, monitoring and accountability for NCD care. Opportunities for further integration include aligning M&E systems to track both HIV and NCD indicators; integrating sample transport for viral load and HbA1c; and coordinating supply chains for antiretrovirals, antihypertensives and diabetes medications. These synergies align with universal health coverage and One Health approaches, potentially reducing costs and improving efficiency.

A critical distinction emerged from the literature: multi‐month prescribing (clinician authorizing 3–6 months of medication) was often possible, but multi‐month dispensing (patient receiving that supply) was frequently restricted by pharmacy stock limits or payment requirements. Separating these two functions can reduce visit frequency even when supply chains are weak, as demonstrated in decentralized adherence club models.

### 4.3 Service Integration, Stigma and Confidentiality

Integrated care models were generally well accepted by patients and providers and were associated with improved appointment adherence and reduced duplication of visits [15, 22]. However, evidence regarding stigma reduction was mixed. While some studies reported decreased HIV‐related stigma, others identified ongoing concerns related to privacy and confidentiality within co‐located services [15, 16].

These findings suggest that physical integration alone is insufficient to address deep‐seated stigma and may introduce new risks, particularly for patients wishing to maintain confidentiality regarding their HIV status. Thus, integration designs must incorporate privacy safeguards, optional service pathways and targeted anti‐stigma interventions rather than relying solely on co‐location.

### 4.4 The Acceptability‐Sustainability Paradox

Our review reveals a critical tension: integrated care models are highly acceptable to patients and providers due to convenience and perceived reduced stigma, yet they face significant systemic barriers to sustainability, including donor dependency, fragile supply chains and workforce shortages [7, 21].

This acceptability‐sustainability paradox suggests that the most patient‐centred models may be the most vulnerable to health system weaknesses. The sustainability conclusions differ substantially by model type. Integrated NCD care within donor‐funded vertical HIV programmes faces high sustainability risk when funding shifts. In contrast, integration within general PHC chronic care clinics (e.g. the INTE‐AFRICA model [4]) may be more sustainable, as it aligns with routine Ministry of Health budgets and serves all chronic conditions regardless of HIV status. Therefore, scaling up integration requires parallel investments in system strengthening, domestic financing and reliable commodity supply chains to ensure that popular models are also durable.

### 4.5 A Tiered Integration Framework for Context‐Adapted Implementation

**FIGURE 2:**
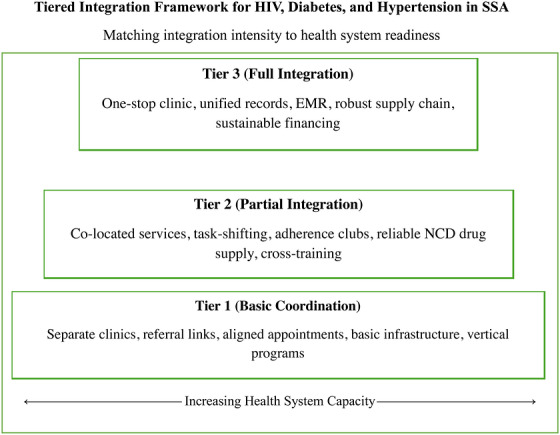
Tiered integration framework for HIV, diabetes and hypertension in SSA.

In response to the heterogeneous health system capacity across SSA, this review proposes a *Tiered Integration Framework* (Figure 2) that aligns the intensity of integration with system readiness. Rather than advocating a single model of full integration, the framework conceptualizes integration as a progressive continuum.

Tier 1 (basic coordination) emphasizes functional linkages between vertical programmes through aligned appointments, referral pathways and shared guidelines [13, 20]. Tier 2 (partial integration) involves co‐located services, task‐shifting and shared patient tracking supported by NCD drug supply and cross‐training [6, 16, 21].

This tier aligns with the most reported models in our review, such as hybrid clinics and adherence clubs, which leverage existing HIV infrastructure while beginning to integrate NCD management. Tier 3 (full integration) represents comprehensive, one‐stop chronic care with unified staffing, records and decentralized drug distribution [4, 14].

This tiered approach provides a pragmatic roadmap for policymakers and implementers, enabling incremental progress while mitigating the risk of overextension in resource‐limited settings. Importantly, it emphasizes the need for parallel investment in NCD‐specific components at each tier to avoid reinforcing HIV‐centred.

### 4.6 Limitations of the Review

Several limitations should be considered. First, only 16 studies met inclusion criteria, and the evidence base is dominated by a small number of research groups and countries, particularly South Africa and Tanzania. This introduces potential for publication and geographic bias, and findings may not be generalizable to all SSA settings, particularly West and Central Africa or fragile states.

Second, the geographic concentration of evidence in Eastern and Southern Africa, settings with longstanding PEPFAR investments in HIV infrastructure, limits generalizability. The proposed tiered integration framework builds on this legacy and may be less directly applicable in settings without robust HIV platforms.

Third, most included studies were clinical trials or well‐supported cohort studies, which may not fully represent routine practice conditions where staffing, supplies and supervision are less consistent. Real‐world effectiveness may be lower than reported efficacy.

Fourth, heterogeneity in study designs, interventions and outcome measurements precluded meta‐analysis and limited direct comparability. Publication bias may favour reporting of successful integration initiatives, potentially under‐representing unsuccessful or stalled efforts. Evidence on long‐term sustainability, cost‐effectiveness and paediatric populations remain limited.

Despite these limitations, this review provides a comprehensive synthesis and identifies critical gaps for future research, particularly in areas of cost‐effectiveness, long‐term sustainability and equity‐focused implementation.

### 4.7 Implications for Policy, Practice and Future Research

**For Policy**: Adopt a differentiated, tiered approach to integration that is adaptable to local system capacity. Move beyond the “HIV‐first” model by mandating parallel investments in NCD medicines, training and monitoring.

**For Practice**: Implement task‐shifting, community‐based drug distribution and simplified monitoring protocols to expand reach and efficiency. Design services with privacy in mind and actively address stigma through patient education and provider training.

**For Research**: Future research should prioritize mixed‐methods evaluations that examine implementation process, cost‐effectiveness and patient‐reported outcomes across diverse settings, with particular attention to how integration affects equity and long‐term clinical outcomes for all conditions.

## 5 Conclusions

Integrated care for HIV, diabetes and hypertension in SSA effectively improves retention in care and viral suppression, yet clinical control of NCDs remain suboptimal. Successful integration requires moving beyond leveraging HIV platforms alone to actively strengthen NCD‐specific components, particularly reliable drug supply chains, workforce training and patient self‐management support. The proposed Tiered Integration Framework offers a scalable, context‐adapted strategy to match integration intensity with local health system readiness. Sustainable scale‐up will depend on policy commitment, domestic financing and systemic investments that ensure integrated care delivers high‐quality outcomes for all chronic conditions.

## Author Contributions

**Dereje Haile**: conceptualization, writing – original draft, validation, visualization, supervision, data curation, software, methodology. **Temesgen Anjulo Ageru**: conceptualization, investigation, funding acquisition, writing – original draft, methodology, software, formal analysis, project administration, data curation, resources, writing – review and editing. **Hien Thi Nguyen**: methodology, visualization, writing – review and editing, writing – original draft, project administration, supervision, data curation, software. **Charuai Suwanbamrung**: conceptualization, writing – original draft, methodology, formal analysis, data curation, resources, project administration, writing – review and editing, investigation. **Esayas Aydiko Amele**: conceptualization, methodology, formal analysis, supervision, validation, software, data curation.

## Funding

This research is financially supported by Walailak University.

## Conflicts of Interest

The authors declare no conflicts of interest.

## Supporting information



Supplementary Material_1 Search Strategies

## Data Availability

All necessary data relevant to this study are included in the manuscript and uploaded as Supplementary Information.
