# Supplementary material for: Integrated Care Models for HIV, Diabetes and Hypertension in Sub‐Saharan Africa: A Systematic Review of Effectiveness, Implementation and Real‐World Applicability
Source: J Int AIDS Soc. 2026 Jul 5;29(7):e70159. doi: 10.1002/jia2.70159 (PMC13334126; doi:10.1002/jia2.70159)
Supplement: Supplementary file 1 — Supplementary Material_1 Search Strategies [file JIA2-29-e70159-s001.pdf]

Supplementary Material_1 Search Strategies

PubMed search strategy

("HIV"[Mesh] OR "HIV Infections"[Mesh] OR hiv[tiab] OR "human immunodeficiency virus"[tiab] OR "people living with HIV"[tiab] OR PLHIV[tiab]) AND ("Diabetes Mellitus"[Mesh:noexp] OR "Hypertension"[Mesh:noexp] OR diabetes[tiab] OR hypertension[tiab] OR "diabetes mellitus"[tiab] OR "high blood pressure"[tiab] OR "non-communicable disease*"[tiab] OR NCD[tiab] OR NCDs[tiab] OR multimorbidity[tiab] OR "multiple chronic conditions"[tiab]) AND ("Delivery of Health Care, Integrated"[Mesh] OR "integrated care"[tiab] OR "integrated model"[tiab] OR "integrated models"[tiab] OR "one-stop"[tiab] OR "task shift*"[tiab] OR "task shar*"[tiab] OR "nurse-led"[tiab] OR decentrali*[tiab] OR "adherence club"[tiab] OR "adherence clubs"[tiab] OR "chronic care"[tiab] OR "collaborative care"[tiab] OR "shared care"[tiab]) AND ("Africa South of the Sahara"[Mesh] OR "sub-Saharan Africa"[tiab] OR "Sub Saharan Africa"[tiab] OR "South Africa"[tiab] OR Tanzania[tiab] OR Kenya[tiab] OR Uganda[tiab] OR Ethiopia[tiab] OR Zimbabwe[tiab] OR Malawi[tiab] OR Eswatini[tiab] OR Swaziland[tiab] OR Botswana[tiab] OR Zambia[tiab] OR Nigeria[tiab] OR Ghana[tiab] OR Rwanda[tiab] OR Mozambique[tiab]) AND (("2016/01/01"[Date - Publication] : "2026/06/01"[Date - Publication]))

Scopus database search strategy

TITLE-ABS-KEY (HIV OR "human immunodeficiency virus" OR PLHIV)

AND TITLE-ABS-KEY (diabetes OR hypertension OR NCD OR NCDs OR multimorbidity)

AND TITLE-ABS-KEY ("integrated care" OR "one-stop" OR "task shift*" OR "nurse-led" OR decentrali* OR "adherence club")

AND TITLE-ABS-KEY ("sub-Saharan Africa" OR "south africa" OR tanzania OR kenya OR uganda OR ethiopia OR zimbabwe OR malawi OR eswatini OR nigeria OR ghana)

AND PUBYEAR > 2015 AND PUBYEAR < 2026

CINAHL

Final search date June 3, 2026

((MH "HIV+") OR (MH "HIV Infections+") OR

TI (hiv OR "human immunodeficiency virus" OR aids) OR AB (hiv OR "human immunodeficiency virus" OR aids))AND ((MH "Diabetes Mellitus+") OR (MH "Noncommunicable Diseases") OR TI (diabet* OR ncd OR "noncommunicable disease*" OR hba1c) OR AB (diabet* OR ncd OR "noncommunicable disease*" OR hba1c))AND ((MH "Delivery of Health Care, Integrated") OR (MH "Primary Health Care") OR (MH "Chronic Disease Management") OR TI ("integrated care" OR "collaborative care" OR "shared care" OR "one-stop" OR "task shift*") OR AB ("integrated care" OR "collaborative care" OR "shared care" OR "one-stop" OR "task shift*")

)AND ((MH "Africa South of the Sahara") OR TI ("sub-saharan africa" OR "subsaharan africa" OR angola OR ethiopia OR kenya OR nigeria OR "south africa" OR tanzania OR uganda OR zimbabwe OR malawi) OR AB ("sub-saharan africa" OR "subsaharan africa" OR angola OR ethiopia OR kenya OR nigeria OR "south africa" OR tanzania OR uganda OR zimbabwe OR malawi))

Web of Science

TS=(hiv OR "human immunodeficiency virus" OR aids) AND TS=(diabet* OR "noncommunicable disease*" OR ncd OR hba1c)

AND TS=("integrated care" OR "collaborative care" OR "shared care" OR "one-stop" OR "task shift*" OR "chronic care model")

AND TS=("sub-saharan africa" OR "subsaharan africa" OR angola OR ethiopia OR kenya OR nigeria OR "south africa" OR tanzania OR uganda OR zimbabwe OR malawi OR zambia OR botswana OR namibia OR mozambique OR rwanda OR eswatini)

Gray literature and hand search

("integrated care" OR "one-stop clinic" OR "task shifting" OR "adherence club") AND (HIV OR diabetes OR hypertension) AND ("sub-Saharan Africa" OR Tanzania OR Kenya OR Uganda OR Ethiopia OR South Africa)
